# Editorial: Advances in T Cell Therapeutic Vaccines for HIV

**DOI:** 10.3389/fimmu.2022.905836

**Published:** 2022-04-27

**Authors:** Bernard J. C. Macatangay, Alan L. Landay, Felipe Garcia, Charles R. Rinaldo

**Affiliations:** ^1^Department of Medicine, University of Pittsburgh School of Medicine, Pittsburgh, PA, United States; ^2^Department of Internal Medicine, Rush Medical College, Chicago, IL, United States; ^3^Infectious Diseases Department, Hospital Clinic Barcelona, Barcelona, Spain

**Keywords:** HIV, therapeutic vaccine, T cell, HIV remission, immunotherapy

Despite significant advancements in our understanding of HIV persistence and immunology, long-term remission of HIV off antiretroviral therapy (ART) has not been achieved except in a few reported cases (1–3). Various strategies aiming to boost immune responses against the virus are currently being evaluated in several proof-of-concept studies and randomized clinical trials. These strategies include harnessing the activity of broadly neutralizing antibodies (bNAbs) (4), innate immune responses (5), and immune checkpoint inhibitors (6, 7). Although newer and more innovative immunotherapeutic approaches are constantly being introduced and evaluated in the HIV cure field, the strategy of therapeutic vaccination to enhance HIV-specific T cell immune responses has been around for over two decades ago and multiple clinical trials have assessed the efficacy of various iterations of these vaccines (8, 9). In general, these vaccines have been safe and well-tolerated, and some of the vaccine approaches had promising results with regard to increasing HIV-specific T cell immune responses. However, despite these results, the overall goal of ART-free HIV remission has remained elusive. The disconnect between clinical outcomes and assay results stress the importance of investigating different factors that could play a role in the success of therapeutic T cell vaccines and is the focus of this Research Topic. Investigating these factors continue to be an important priority in HIV cure research.

As it is with preventative vaccine research, the specific vaccine type constitutes an important factor that can contribute to the safety, tolerability, and efficacy of the vaccine. Previous studies have used DNA vaccines, viral vectors, inactivated whole virus depleted of gp120, and monocyte-derived autologous dendritic cells (DCs) (10). The strategy of DC-based vaccination capitalizes on the ability of these potent antigen presenting cells to coordinate and enhance T cell immunity and is indeed one of the most promising strategies (11–13). The review by Espinar-Buitrago and Munoz-Fernandez not only elucidates DC-T cell interactions but also strategies to better employ DC function, specifically discussing ways of introducing viral peptides to DCs such as the use of nanoparticles as delivery agents. Meanwhile, the randomized placebo-controlled clinical trial by Leal et al., evaluated the safety, immunogenicity, and virologic effects of three doses of ultrasound-guided intranodal infections of a monocyte-derived DC loaded with autologous heat-inactivated HIV every two weeks with or without 3 weekly doses of subcutaneously administered pegylated interferon (IFN) α-2a, the first being given at the last DC vaccine dose. Participants then stopped ART at the last vaccine dose and were observed for viral rebound. The vaccine and the pegylated IFN α-2a were safe and well-tolerated and generated higher HIV-specific IFNγ ELISPOT responses compared to placebo. The study also showed a very modest decrease in viral load setpoint among vaccine recipients which correlated with the increase in HIV-specific T cell responses. Despite these findings, all participants had viral rebound within the 12-week treatment interruption period with a median time to rebound of 27 days.

Results of this clinical trial highlight the disconnect between the various immunologic and virologic assays used to evaluate vaccine response and the clinical outcome of viral rebound following ART interruption. Although development of innovative ways to optimize the ability of therapeutic vaccines to enhance cellular immunity is important, addressing this disconnect remains to be a critical gap in our understanding of this vaccination strategy. This would involve identifying which specific combinations of immunologic and virologic parameters would best predict time to viral rebound. This is one of the main objectives of the AIDS Clinical Trials Group (ACTG) study A5345 (14). Results of that study will be helpful in designing future clinical studies. In addition, developing newer highly reproducible assays that could provide immunologic readouts that significantly correlate with viral rebound is paramount so these could be used in therapeutic vaccine trials. In the paper by Xu et al., the investigators address the substantial variability observed in viral inhibition assays and describe ways by which they optimized the 7-day protocol. The modifications allowed improved reproducibility and also resulted in a larger assay dynamic range to detect % inhibition which could be beneficial in identifying changes in therapeutic vaccine trials. Interestingly, there was no correlation between this assay and magnitude of T cell responses measured using IFNγ ELISPOT or cytolytic function using intracellular cytokine staining, two assays commonly used in vaccine trials. It would be important to assess the ability of this assay to predict rebound in completed or ongoing vaccine trials where participants undergo treatment interruption.

Finally, one approach to further enhancing immune responses against HIV is to employ several arms of the immune system. This immunotherapeutic strategy is currently being investigated in a number of ongoing clinical trials and studies in development. The study by Veenhuis et al. provide the supporting rationale for this approach. In their paper, they describe the development of an assay that can measure effector functions of CD8+ T cells and bNAbs. The assay involves co-culture of isolated CD4+ T cells with HIV and bNAb immune complexes, infection of CD4+ T cells by spinoculation, and a suppression assay with CD8+ T cells. The authors show through this *in vitro* model that CD8+ T cells and bNAbs act synergistically in inhibiting HIV. This vaccinal effect of bNAb therapy has indeed been demonstrated in both nonhuman primate studies and clinical trials (15, 16). A study in development within the ACTG evaluating therapeutic vaccination together with bNAb therapy and which includes a treatment interruption phase will be crucial in determining whether this approach will indeed result in HIV remission. Other combination therapies which would include innate immune responses or strategies to reverse latency are also being evaluated in different clinical trials. These studies highlight the fact that since HIV pathogenesis affects various arms of the immune system, immunotherapeutic interventions targeting these different arms may be necessary in achieving the goal of long-term HIV remission off ART.

## Author Contributions

BM, wrote editorial, edited; AL, edited; FG, edited; CR, edited. All authors contributed to the article and approved the submitted version.

## Funding

This publication is supported in part by the National Institutes of Health Cooperative Agreement U01-HL146203 and U01-AI131285, and by INT21/00033, Spanish Ministry of Science RTI2018-096309-B-I00, HIVACAR H2020-SC1-2016-2017 (H2020-SC1-2016-RTD) Proposal 731626.

## Conflict of Interest

The authors declare that the research was conducted in the absence of any commercial or financial relationships that could be construed as a potential conflict of interest.

## Publisher’s Note

All claims expressed in this article are solely those of the authors and do not necessarily represent those of their affiliated organizations, or those of the publisher, the editors and the reviewers. Any product that may be evaluated in this article, or claim that may be made by its manufacturer, is not guaranteed or endorsed by the publisher.

## References

[1] Allers K Hutter G Hofmann J Loddenkemper C Rieger K Thiel E. Evidence for the Cure of HIV Infection by CCR5Delta32/Delta32 Stem Cell Transplantation. Blood (2011) 117:2791–9. doi: 10.1182/blood-2010-09-309591 21148083

[2] Gupta RK Abdul-Jawad S McCoy LE Mok HP Peppa D Salgado M. HIV-1 Remission Following CCR5Delta32/Delta32 Haematopoietic Stem-Cell Transplantation. Nature (2019) 568:244–8. doi: 10.1038/s41586-019-1027-4 PMC7275870 30836379

[3] Hutter G Nowak D Mossner M Ganepola S Mussig A Allers K. Long-Term Control of HIV by CCR5 Delta32/Delta32 Stem-Cell Transplantation. N Engl J Med (2009) 360:692–8. doi: 10.1056/NEJMoa0802905 19213682

[4] Caskey M. Broadly Neutralizing Antibodies for the Treatment and Prevention of HIV Infection. Curr Opin HIV AIDS (2020) 15:49–55. doi: 10.1097/COH.0000000000000600 31764199 PMC7340121

[5] Board NL Moskovljevic M Wu F Siliciano RF Siliciano JD. Engaging Innate Immunity in HIV-1 Cure Strategies. Nat Rev Immunol (2021). doi: 10.1038/s41577-021-00649-1 34824401

[6] Gay CL Bosch RJ McKhann A Moseley KF Wimbish CL Hendrickx SM. Suspected Immune-Related Adverse Events With an Anti-PD-1 Inhibitor in Otherwise Healthy People With HIV. J Acquir Immune Defic Syndr (2021) 87:e234–6. doi: 10.1097/QAI.0000000000002716 PMC8263135 33929394

[7] Gay CL Bosch RJ Ritz J Hataye JM Aga E Tressler RL. Clinical Trial of the Anti-PD-L1 Antibody BMS-936559 in HIV-1 Infected Participants on Suppressive Antiretroviral Therapy. J Infect Dis (2017) 215:1725–33. doi: 10.1093/infdis/jix191 PMC5790148 28431010

[8] Garcia F Leon A Gatell JM Plana M Gallart T. Therapeutic Vaccines Against HIV Infection. Hum Vaccin Immunother (2012) 8:569–81. doi: 10.4161/hv.19555 22634436

[9] Stephenson KE. Therapeutic Vaccination for HIV: Hopes and Challenges. Curr Opin HIV AIDS (2018) 13:408–15. doi: 10.1097/COH.0000000000000491 29957615

[10] Gray GE Laher F Lazarus E Ensoli B Corey L. Approaches to Preventative and Therapeutic HIV Vaccines. Curr Opin Virol (2016) 17:104–9. doi: 10.1016/j.coviro.2016.02.010 PMC5020417 26985884

[11] Garcia F Plana M Climent N Leon A Gatell JM Gallart T. Dendritic Cell Based Vaccines for HIV Infection The Way Ahead. Hum Vaccin Immunother (2013) 9:2445–52. doi: 10.4161/hv.25876 PMC3981855 23912672

[12] Rinaldo CR. Dendritic Cell-Based Human Immunodeficiency Virus Vaccine. J Intern Med (2009) 265:138–58. doi: 10.1111/j.1365-2796.2008.02047.x PMC2875880 19093966

[13] Ko EJ Robert-Guroff M. Dendritic Cells in HIV/SIV Prophylactic and Therapeutic Vaccination. Viruses (2019) 12. doi: 10.3390/v12010024 PMC7019216 31878130

[14] Li JZ Aga E Bosch RJ Pilkinton M Kroon E MacLaren L. Time to Viral Rebound After Interruption of Modern Antiretroviral Therapies. Clin Infect Dis (2022) 74:865–70. doi: 10.1093/cid/ciab541 PMC8906742 34117753

[15] Naranjo-Gomez M Pelegrin M. Vaccinal Effect of HIV-1 Antibody Therapy. Curr Opin HIV AIDS (2019) 14:325–33. doi: 10.1097/COH.0000000000000555 30973419

[16] Niessl J Baxter AE Mendoza P Jankovic M Cohen YZ Butler AL. Combination Anti-HIV-1 Antibody Therapy is Associated With Increased Virus-Specific T Cell Immunity. Nat Med (2020) 26:222–7. doi: 10.1038/s41591-019-0747-1 PMC7018622 32015556

